# Monoclonal IgM Antibodies Targeting *Candida albicans* Hyr1 Provide Cross-Kingdom Protection Against Gram-Negative Bacteria

**DOI:** 10.3389/fimmu.2020.00076

**Published:** 2020-02-18

**Authors:** Eman G. Youssef, Lina Zhang, Sondus Alkhazraji, Teclegiorgis Gebremariam, Shakti Singh, Nannette Y. Yount, Michael R. Yeaman, Priya Uppuluri, Ashraf S. Ibrahim

**Affiliations:** ^1^Division of Infectious Diseases, Harbor-UCLA Medical Center, Torrance, CA, United States; ^2^The Lundquist Institute for Biomedical Innovation, Harbor-UCLA Medical Center, Torrance, CA, United States; ^3^Department of Biotechnology and Life Sciences, Faculty of Postgraduate Studies for Advanced Sciences, Beni-Suef University, Beni-Suef, Egypt; ^4^College of Wildlife Resources, Northeast Forestry University, Harbin, China; ^5^Division of Molecular Medicine, Harbor-UCLA Medical Center, Torrance, CA, United States; ^6^Department of Medicine, David Geffen School of Medicine at UCLA, Los Angeles, CA, United States

**Keywords:** monoclonal antibodies, *Candida* Hyr1, *Acinetobacter baumannii*, *Klebsiella pneumoniae*, passive vaccine, molecular modeling, cross-kingdom immunotherapy

## Abstract

Recent years have seen an unprecedented rise in the incidence of multidrug-resistant (MDR) Gram-negative bacteria (GNBs) such as *Acinetobacter* and *Klebsiella* species. In view of the shortage of novel drugs in the pipeline, alternative strategies to prevent, and treat infections by GNBs are urgently needed. Previously, we have reported that the *Candida albicans* hypha-regulated protein Hyr1 shares striking three-dimensional structural homology with cell surface proteins of *Acinetobacter baumannii*. Moreover, active vaccination with rHyr1p-N or passive immunization with anti-Hyr1p polyclonal antibody protects mice from *Acinetobacter* infection. In the present study, we use molecular modeling to guide design of monoclonal antibodies (mAbs) generated against Hyr1p and show them to bind to priority surface antigens of *Acinetobacter* and *Klebsiella pneumoniae*. The anti-Hyr1 mAbs block damage to primary endothelial cells induced by the bacteria and protect mice from lethal pulmonary infections mediated by *A. baumannii* or *K. pneumoniae*. Our current studies emphasize the potential of harnessing Hyr1p mAbs as a cross-kingdom immunotherapeutic strategy against MDR GNBs.

## Introduction

Infections caused by multidrug-resistant organisms (MDROs) pose increasing therapeutic challenges. In the past decade, *Acinetobacter baumannii* has emerged as one of the most common MDROs in hospital-acquired infections, causing a range of diseases from pneumonia to sepsis or wound infections (1–6). Of great concern is that 40–70% of *A. baumannii* isolates are now extensively drug resistant (XDR; i.e., resistant to all antibiotics except colistin or tigecycline), reflecting a >15-fold increase since 2000 (1, 6–8). Likewise, the Enterobacteriaceae organism *Klebsiella pneumoniae* causes high rates of morbidity and mortality in critically ill, hospitalized patients. In recent years, strains of *K. pneumoniae* have exhibited resistance to almost all classes of antibacterial drugs, including carbapenems (9–11). Together, *Acinetobacter* and carbapenem-resistant *K. pneumoniae* (KPC) have been prioritized by the U.S. Centers for Disease Control and Prevention (CDC) as two of the top “serious threat level pathogens” owing to resistance, failure of the current standard of treatment, and high mortality rates. Amplifying these concerns, the existing drug development pipeline against these pathogens is sparse, and it is almost certain that these organisms will develop resistance to any future approved antibiotics. Hence, novel strategies to prevent and treat life-threatening infections caused by these and related MDRO pathogens are urgently needed.

We previously developed innovative computational molecular modeling and bioinformatics strategies to discover novel vaccine and immunotherapy candidates targeting more than one high-priority pathogen. The application of this methodology has been used to successfully discover and develop novel cross-kingdom vaccines (12). Among other advances, this discovery strategy culminated in the identification of *Candida albicans* Hyr1p, a hypha-regulated cell surface protein. Although Hyr1p is strictly expressed on *C. albicans* hyphae, it has no effect on the fungus germination and subsequent hyphal formation (13). However, we have shown that Hyr1p contributes to *C. albicans* virulence by resisting phagocyte killing (a major host defense mechanism against candidiasis) through a mechanism that is yet to be identified (14). Indeed, mice vaccinated with Hyr1p are protected from *C. albicans* infections (14, 15). Recently, we found that the Hyr1p shares striking three-dimensional (3-D) structural and epitope homologies with antigens present on the Gram-negative bacterium (GNB) *A. baumannii*, including with the putative hemagglutinin/hemolysin protein FhaB, outer membrane protein class A (OmpA), and a number of siderophore-binding proteins (16). All these putative cross-reactive antigens are known contributors to bacterial virulence. Specifically, FhaB and OmpA help in bacterial adhesion and biofilm formation (17–19). Also, OmpA plays a role in conferring multidrug resistance of *A. baumannii* to antibiotics (20). Finally, the *A. baumannii* siderophore acinetobactin was shown to be required for bacterial infection by acquiring iron from the host (21), therefore implicating siderophore receptors in the virulence of the bacterium. Polyclonal antibodies (pAbs) raised against peptides derived from the Hyr1p N-terminus blocked *A. baumannii-*mediated lung epithelial cell damage and killed the bacterium *in vitro* (16). Importantly, anti-Hyr1p pAbs completely protected mice from *A. baumannii* infections. These results provided compelling proof of concept for targeting Hyr1p for developing immunotherapies against GNBs and laid a groundwork for generation and evaluation of the efficacy of anti-Hyr1p monoclonal antibodies (mAbs) targeting MDR GNBs.

In the current study, we generated mAbs against peptide #5 of Hyr1 and affirm that these mAbs not only recognize different clinical isolates of *A. baumannii* but also bind to drug-resistant *K. pneumoniae*. We further demonstrate the efficacy of these targeted mAbs in blocking bacterial-mediated host cell damage and in protecting mice against lethal pulmonary infection by both MDR bacteria. Given that there are currently no immunotherapies against GNBs and the alarming rate at which MDROs are increasing as a global threat to public health, active, or passive vaccination strategies using vaccines or mAbs, respectively, are now highly attractive immunotherapeutic modalities to prevent or treat these refractory infections either as standalone or antibiotic-adjunctive therapies.

## Results

### Hyr1p Is Structurally Homologous to Target Surface Antigens of *Klebsiella pneumoniae*

**Figure 1:**
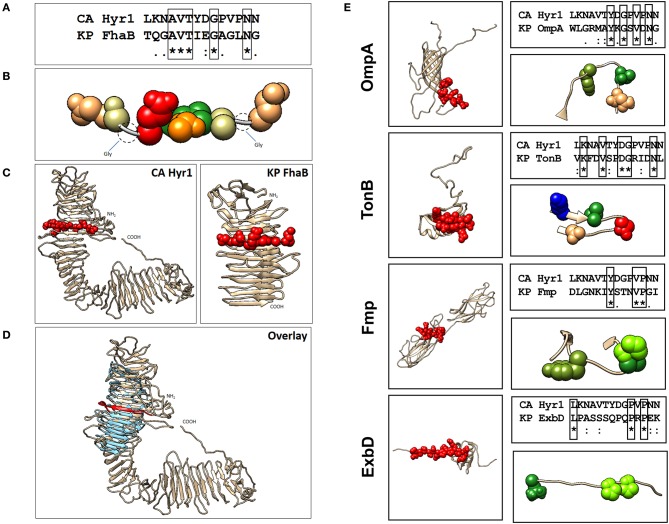
Localization of Hyr1 peptide #5 and putative cross-reactive epitopes in modeled *Klebsiella pneumoniae* target sequences. **(A)** Sequence alignments between Hyr1 peptide #5 and putative cross-reactive motifs within *K. pneumoniae* FhaB sequence; identical residues are boxed. **(B)** van der Waals space-filling models illustrating conservation of amino acid physicochemistry in the highly homologous motifs; coloration is a modified RasMol schema (Gly, Ala–cream; Asn, Gln–khaki; Thr–orange; Val–green; Asp–red). **(C)** Comparative models of *Candida albicans* Hyr1 and *K. pneumoniae* FhaB showing homologous cross-reactive domains in red van der Waals space-filling spheres within the homologous domains of the two proteins. **(D)** Superimposition overlay of the homologous regions of Hyr1 and FhaB showing strong 3-D homology in antiparallel β-sheet facets and overall structures in which the conserved target motifs exist. Peptide #5 is shown in red as van der Waals space-filling spheres in the comparative Hyr1 and FhaB models. **(E)** Individual models for four targeted cross-reactive antigens of *K. pneumoniae* (OmpA, TonB, Fmp, and ExbD) are shown with domains homologous to Hyr1 peptide #5 in red. Sequence alignments between Hyr1 peptide #5 and cross-reactive target motifs with identical residues are boxed; also shown are domains showing identical and/or physicochemically conserved residues as space-filling spheres; coloration is a modified RasMol schema (Gly, Ala–cream; Asn, Gln–khaki; Thr, Ser–orange; Val, Ile, Leu, Met, Cys–green; Trp, Tyr, Phe–olive green; Asp, Glu–red; Arg, Lys–blue; His–sky blue; Pro–chartreuse).

Our previous studies involving complimentary homology and energy-based modeling algorithms identified structural domains conserved between Hyr1p and the GNB *Acinetobacter baumannii* (16). We questioned if other GNBs could similarly share conserved physiochemical structural domains. Of great relevance, we identified strong homology between Hyr1p and filamentous hemagglutinin B (FhaB) of *Klebsiella pneumoniae* (Figures 1A–D). This highly conserved homology was reflected at the level of amino acid sequences in a shared target motif (Figures 1A–B), relative structural integration of this motif in the larger holoproteins (Figure 1C), and overall 3-D homology of the two proteins (Figure 1D). Furthermore, our modeling studies revealed four other proteins in *K. pneumoniae* that displayed conserved 3-D homology with Hyr1p: OmpA, transporter of nutrients B (TonB), fimbrial protein (Fmp), and the biopolymer export protein D (ExbD). Following energy minimization and hydrogen-bond optimization to yield 3-D structure models, these proteins were aligned further with a 14-amino-acid peptide of Hyr1p (LKNAVTYDGPVPNN; also called peptide #5)–a highly antigenic, surface-exposed domain of the protein, for which anti-peptide pAbs were shown to protect against murine *A. baumannii* infection (16). This was done to localize specific homology sites hypothesized to confer protective efficacy (Figures 1A–D). Modeling data demonstrated that the identified 3-D structures corresponded with a conserved sequence region within each target antigen (Figure 1E). Based on strong efficacy seen in cross-kingdom immunization studies of prior modeling-predicted antigens (e.g., Hyr1p vs. *A. baumannii*) (16), the current model predictions were interpreted as supporting confidence in cross-protective efficacy against *K. pneumoniae*.

### Anti-Hyr1 Monoclonal Antibodies Bind to Gram-Negative Bacteria

**Figure 2:**
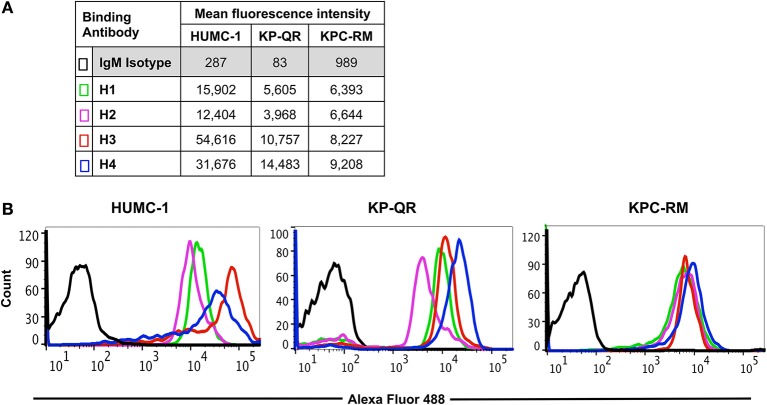
Binding of monoclonal antibodies (mAbs) targeting Hyr1 peptide #5 to Gram-negative bacteria (GNBs). MAb clones (and isotype-matched control) IgM were evaluated for binding to *Acinetobacter baumannii* (HUMC-1), *Klebsiella pneumoniae*-QR (KP-QR), and *K. pneumoniae*-RM (KP-RM) at a concentration of 100 μg/ml. The extent of binding was quantified by flow cytometry after staining the bound antibodies with Alexa 488-conjugated secondary antibody. Data were represented as mean fluorescence intensity of the Ab-bound bacteria **(A)**. The degree of binding was also visualized by a shift in the peaks in the anti-Hyr1 IgM binding conditions vs. the control antibodies **(B)**.

We previously reported that pAbs raised against Hyr1 peptide #5 blocked virulence functions of *A. baumannii in vitro* and completely protected diabetic and neutropenic mice from *Acinetobacter* bacteremia and pulmonary infection, respectively (16). Encouraged by these results, and to enhance the therapeutic potential of such antibodies, we developed mAbs against the same surface-exposed and immunodominant peptide (peptide #5). These mAbs (all IgM isotypes) were tested for their abilities to bind to *Candida albicans* as well as the GNB *A. baumannii* or *K. pneumoniae*. Four individual fluorescein isothiocyanate (FITC)-labeled mAb clones (H1, H2, H3, and H4; 100 μg/ml) were tested against three prototypic MDR GNB strains, including *A. baumannii* (HUMC-1, XDR clinical isolate); *K. pneumoniae*-RM (KPC-RM, carbapenem-resistant clinical isolate); and *K. pneumoniae*-QR (KP-QR, MDR strain sensitive to carbapenem). The extent of mAb binding to each of the pathogen surfaces, as compared with isotype-matched non-specific control antibodies, was then quantified by flow cytometry. Of the four mAb clones tested, compared with the isotype-matched control IgM, H3, and H4 displayed the highest levels of binding to all GNBs, with at least 10–300-fold increases (Figure 2A). Binding potential was also visualized by a shift in the peaks of the anti-Hyr1p IgM binding vs. the isotype-matched control antibodies (Figure 2B). The right shift in the peaks of individual mAb also correlated with their respective increase in mean fluorescence of the cells.

**Figure 3:**
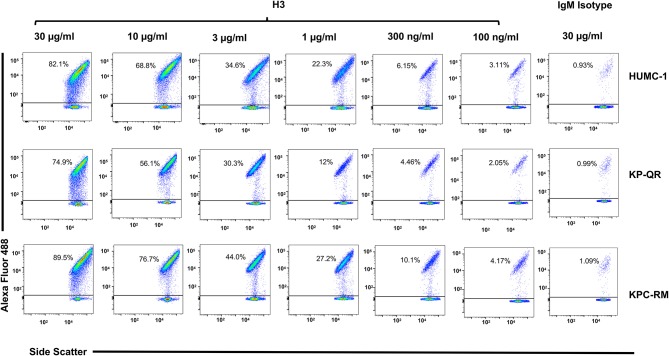
Monoclonal antibody (mAb) clone H3 targeting Hyr1 peptide #5 binds Gram-negative bacteria (GNBs) in a dose-dependent manner. MAb clone H3 (and IgM isotype control) were evaluated for binding to HUMC-1, KP-QR, and KP-RM at concentrations ranging from 30 to 0.1 μg/ml. The extent of binding was quantified by flow cytometry after staining the antibodies with Alexa 488-conjugated secondary antibody. Data were represented in a scatter plot highlighting the percentage of bacteria that were bound by the Abs.

Next, we compared the relative binding ability of the mAb clones to recognize the distinct GNBs. Clone H3 bound to all three organisms even at low mAb concentrations. Specifically, 30 μg/ml of H3 mAb bound 82, 75, and 90% of *A. baumannii* HUMC-1, KP-QR, and KPC-RM cells, respectively, whereas the isotype-matched control did not bind to any of the bacterial cells (<1%). The binding of the H3 mAbs to either *A. baumannii* or *K. pneumoniae* was maintained even at a very low concentration of 300 ng/ml, demonstrating 4–10-fold increase over the binding ability of the isotype-matched control IgM (Figure 3). Similarly, the H4 clone bound to each of the GNBs at 30 μg/ml of concentration (data not shown). We further evaluated the binding of the two clones H3 and H4 against other drug-resistant clinical isolates of *A. baumannii* and *K. pneumoniae* (KPC). The mAbs bound HUMC-6, HUMC-12, KPC-6, and KPC-8 at significantly higher capacity as than does the control IgM (Supplementary Figure 1). These results indicate that binding of mAbs to the surface of the target GNBs is not isolate specific, supporting our consensus epitope hypothesis.

### Monoclonal Antibodies Protect Host Cells From Damage by Gram-Negative Bacteria

**Figure 4:**
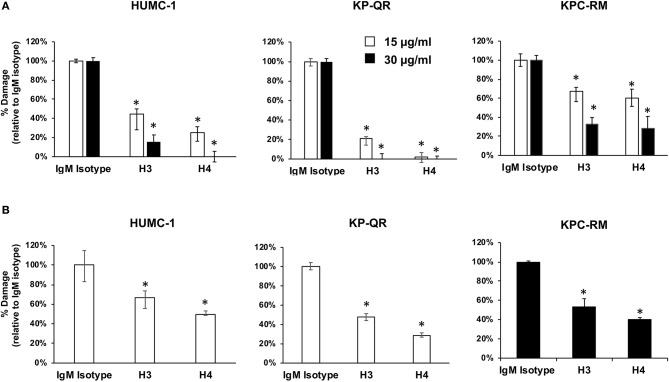
Monoclonal antibodies (mAbs) targeting Hyr1 peptide #5 prevents HUMC-1-, KP-QR-, and KP-RM-induced A549 lung alveolar epithelial cells and primary human umbilical vein endothelial cells (HUVECs) damage. HUMC-1-, KP-QR-, and KPC-RM-induced A549 cell injury in the presence of 15 or 30 μg/ml of an isotype-matched IgM, mAb H3, or mAb H4 **(A)**. Damage to HUVECs by HUMC-1 and KP-QR (in the presence of 15 μg/ml of H3 and H4), also KPC-RM (30 μg/ml of the mAbs) **(B)**. Cell damage was determined by ^51^Cr-release assay after 48 and 24 h for HUMC-1 and KP, respectively. Percentage damage was normalized to IgM isotype-matched control after subtracting spontaneous cell damage. *P < 0.001 vs. control IgM. *N* = 12 per group from three independent experiments.

Our previous studies demonstrated that anti-Hyr1p pAbs not only bound to *A. baumannii* but also inhibited the ability of bacterium to interact with and damage mammalian cells (16). Thus, we hypothesized that the mAbs would similarly block damage of host cells caused by these GNBs. Concordant with this hypothesis, mAb clones H3 and H4 at 15 or 30 μg/ml blocked the ability of *A. baumannii* (HUMC-1) and *K. pneumoniae* (KP-QR or KPC-RM) to damage A549 lung alveolar epithelial cells. Specifically, both mAbs showed a dose–response inhibition of GNB-mediated A549 cell damage with the 15 μg/ml of dose resulting in 40–90% inhibition and the 30 μg/ml dose causing ~70–100% inhibition (Figure 4A). The two mAbs also protected A549 cells from damage by other clinical isolates of GNBs such as HUMC-6 and KPC-8 (Supplementary Figure 2). Consistent with these results, both mAbs at 15 μg/ml resulted in ~40–70% inhibition of *A. baumannii* HUMC-1- or KP-QR-mediated damage to primary human umbilical vein endothelial cells (HUVECs). However, it took a higher mAb concentration, 30 μg/ml, to protect HUVECs from KPC-RM (Figure 4B). Overall, these results show that mAbs raised against Hyr1 peptide #5 bind to MDR *A. baumannii* and *K. pneumoniae* strains and mitigate the ability of these bacteria to damage host cells *in vitro*.

### Anti-Hyr1 Monoclonal Antibodies Protect Mice From Pulmonary Infection Caused by *Acinetobacter baumannii* or *Klebsiella pneumoniae*

**Figure 5:**
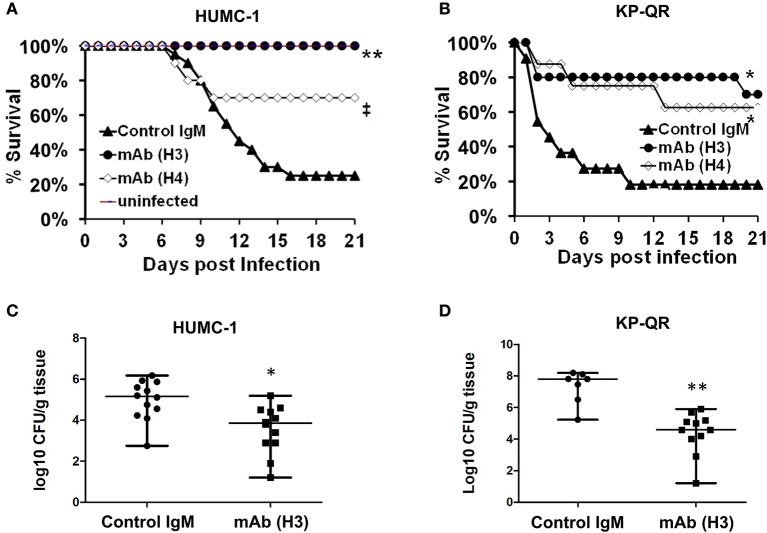
Monoclonal antibody (mAb) clones H3 and H4, targeting Hyr1 peptide #5, protect mice from HUMC-1- or KP-QR- induced pneumonia. Immunosuppressed CD-1 male mice (*n* = 10/group from two experiments) were infected with HUMC-1 *via* inhalation [average 5 × 10^10^ colony-forming units (CFU)] **(A)**. Immunocompetent mice (*n* = 10/group from two experiments) were infected intratracheally with KP-QR (average 3.4 × 10^7^ CFU) **(B)**. Intraperitoneal (i.p.) treatment with mAb H3, H4, or isotype-matched control IgM started 24 h and repeated at 96 h post infection (30 μg/mouse each dose). *P < 0.05, *P* = 0.06, and **P < 0.001 vs. control IgM by log-rank test. For CFU measurement, H3 and control IgM were administered 6 h and 3 days post infection, and lungs harvested from mice at Day +4 (for HUMC-1) **(C)** and at Day +2 (for KP-QR) post infection **(D)**.

We tested the ability of the mAbs, given their efficacy in reducing GNB-induced host cell damage *in vitro*, to protect mice from GNB infection. Pneumonia is a life-threatening manifestation of the disease caused by both *A. baumannii* and *K. pneumoniae* (22–25). Thus, we evaluated H3 and H4 for their ability to protect against such infections caused by *A. baumannii* HUMC-1. Although benign in immunocompetent individuals, *A. baumannii* can cause life-threatening pneumonia in immunosuppressed hospitalized patients (25). Thus, we additionally evaluated the efficacy of mAb therapy in a neutropenic mouse model infected with HUMC-1 *via* inhalation. The mAbs were administered intraperitoneally (i.p.) at a dose of 30 μg/mouse, in established infection on Days +1 and +4 relative to infection. Placebo mice were treated in an identical regimen with an isotype-matched control IgM. Treatment with mAb H4 yielded a high (70%) overall survival, vs. 20% overall survival for control IgM treatment (*P* < 0.06). Impressively, complete protection (100% survival) was conferred in mice receiving H3 mAb treatment, *P* < 0.001 (Figure 5A). Surviving mice appeared healthy on Day +21 post infection, when the experiment was terminated.

We next evaluated the efficacy of mAbs in a similar mouse model of *K. pneumoniae* pulmonary infection. Our *in vivo* optimization studies showed that KP-QR exhibits pronounced lethality even in healthy immune competent mice (Supplementary Figure 3A), whereas KPC-RM is avirulent despite high inocula used for infection (Supplementary Figure 3B). Thus, we evaluated the protective effect of mAbs against the KP-QR-mediated pneumonia in mice. Immunocompetent mice were infected intratracheally with KP-QR and treated twice as above with either the H3 or H4 mAbs, or isotype-matched control IgM. Almost 60% of mice treated with H4 survived the otherwise lethal challenge by KP-QR (*P* < 0.05). Consistent with protection against host cell damage, even greater efficacy was observed with mAb H3, which exhibited protection trending to 80% survival vs. 20% survival in mice treated with isotype-matched IgM (*P* < 0.05) (Figure 5B). Surviving mice appeared healthy at 21 days post infection at the experimental endpoint.

We also performed studies to assess the effect of the mAb treatment on the bacterial burden in lung tissues, along with survival efficacy. Mice were infected as above and treated with H3 mAb once at 6 h post infection for KP-QR and twice (6 h and a repeat dose on Day +3 post infection) for *A. baumannii* HUMC-1. Mice were sacrificed on Day +2 for KP-QR and Day +4 for HUMC-1, and the lungs were harvested for bacterial burden enumeration by quantitative culture. Corroborating the survival data and in comparison with treatment with isotype-matched IgM control, H3 mAb treatment resulted in 1.5- or 3-log reductions in lung bacterial burden of HUMC-1 (*P* < 0.01) or KP-QR (*P* < 0.001), respectively (Figure 5C).

Together, these results demonstrate that therapeutic mAbs derived from innovative methods to exploiting cross-kingdom epitope homology exhibit striking efficacy in life-threating GNB infection. These results emphasize the strong proof-of-concept translational potential to develop such agents as novel therapeutic modalities for prevention or treatment of infections due to MDR GNBs in immunocompetent as well as immunosuppressed or immunodeficient patients.

## Discussion

Phylogenetically diverse pathogens may exploit common host settings and rely on convergent virulence strategies (e.g., cell adhesion, invasion, and injury). Indeed, the fungus *Candida albicans* and certain GNBs, such as *Acinetobacter baumannii* and *Klebsiella pneumoniae*, infect similar immunocompromised, burn, and surgical wound patients in intensive care units (ICUs) or otherwise hospitalized (7, 19, 26). Interestingly, *Candida* species colonization among ICU patients have been identified as an independent risk factor for development of *A. baumannii* ventilator-associated pneumonia (27). Similarly, *Candida* and *Klebsiella* are the most frequent pathogens of the respiratory tract of patients with chronic obstructive pulmonary disease (COPD) (28, 29). Independent of such an association, *Candida* and GNBs individually cause healthcare-associated infections, often leading to significant morbidity and mortality. As a group, GNBs in particular, including *A. baumannii* and *K. pneumoniae*, have evolved into MDR pathogens that cause infections that are often incurable (30). Hence, novel approaches that address antibiotic resistance and leverage or amplify immune function represent highly logical strategies to combat this resistance crisis.

Our group has developed advanced computational, molecular modeling, and bioinformatics strategies to discover novel vaccine antigen candidates that leverage the concept of convergent immunity to target more than one high-priority human pathogen (16, 31, 32). This strategy, also known as unnatural or heterologous immunity, has been previously applied in the development of viral and bacterial vaccines in which an antigen protects against another pathogen from the same or from a different kingdom (12, 25). We have previously validated this approach by demonstrating cross-kingdom immuno-protection against *C. albicans* and *Staphylococcus aureus*, in which the *C. albicans* cell surface adhesin/invasion proteins [agglutinin-like sequence (Als) family of proteins] share epitope and functional homology with MSCRAMMs of *S. aureus* (e.g., clumping factor A) (33). A recombinant form of N-terminus of the Als3p (rAls3p-N) elicits robust T- and B-cell responses and protects mice from both *Candida* and methicillin-resistant *S. aureus* (MRSA) infections (32, 34–39). Most recently, we reported that a distinct hyphal cell surface protein of *C. albicans*, Hyr1p, has epitope homologies with candidate antigens of the MDR GNB *A. baumannii* (16). Indeed, with the use of different mouse models, active or passive immunization (with pAbs) targeting either Als3p or Hyr1p protected mice from *S. aureus* or *A. baumannii* infections, respectively (16, 32, 34). In particular, antibodies against one specific surface-exposed and highly antigenic 15-mer peptide of Hyr1 (peptide #5) offered the highest protection to host cells from *A. baumannii* both *in vitro* and *in vivo* (16).

Homology and energy-based modeling was conducted to compare the overall and target motif-specific physicochemical features of Hyr1 protein with candidate *K. pneumoniae* target antigens. These methods predicted Hyr1p to share 3-D and sequence conservation with a number of proteins expressed on the *K. pneumoniae* surface. In addition to FhaB, significant homologies were observed between the Hyr1 peptide #5 domain that induced highly protective antisera, and OmpA, TonB, Fmp, and ExbD common to *K. pneumoniae* and other high-priority GNBs. Encouraged by the potential of the pAbs, and to further the clinical relevance of our studies, we generated mAbs against the highly antigenic peptide #5 of the Hyr1p. Similar to pAbs, the current results demonstrate that the mAbs blocked the MDR *A. baumannii-* or *K. pneumoniae*-mediated host cell damage and protected mice from otherwise lethal pulmonary infections caused by these pathogens. Initial functional assays revealed that four different mAb clones (H1–H4) recognized the two genera of bacteria for *in vitro* binding at low concentrations of the antibodies. This targeting propensity was extended also to include several MDR clinical isolates of *A. baumannii* and *K. pneumoniae*.

The ability of the mAbs (H3 and H4) to block GNB-mediated damage of host cells was more pronounced in *A. baumannii* HUMC-1, *A. baumannii* HUMC-6, and *K. pneumoniae* KP-QR than in KPC-RM or KPC-8. Virulence factors, including capsule, lipopolysaccharide, fimbriae, and siderophores, have been identified as important for the virulence and/or resistance of KP strains to antibiotics (40). Thus, the resistance of KPC strains KPC-RM or KPC-8 to mAbs could conceivably be due to the difference in the exostructure of these organisms [e.g., differences in lipopolysaccharide (LPS) hindrance and reported production of a larger repertoire of siderophores] (40). Consistent with this hypothesis, our recent findings emphasized the importance of anti-Hyr1 peptide #5 pAbs in blocking iron uptake, leading to killing of the GNB *A. baumannii* (16).

Importantly, the mAbs afforded nearly 70% protection from cellular damage by all GNBs tested, supporting their potential use as preventive or therapeutic modalities with a capacity to block virulence of GNBs. Our recent report using bioinformatics, homology, and energy-based modeling strategies established that *C. albicans* Hyr1p shares striking epitope homology to *A. baumannii* FhaB protein, and anti-peptide #5 pAb bound to FhaB as well as two other proteins on *A. baumannii* based on two-dimensional Western blotting assays (16). The other two *A. baumannii* proteins with considerable homology to Hyr1p included the OmpA, and a ferric siderophore outer membrane binding protein (TonB) (16). Not surprisingly, these three proteins are well conserved in *K. pneumoniae* displaying >60% sequence homology [protein sequence National Center for Biotechnology Information (NCBI) blast alignment] and even greater 3-D homology to their *Acinetobacter* counterparts. Whether these proteins have a significant role in virulence or nutrient uptake–and hence blocking their function would contribute to the killing mechanisms afforded by the mAbs–is the subject of ongoing research by our group.

Because the mAbs significantly blocked the capacity of GNBs to damage host cells *in vitro*, we evaluated their potential to protect against lethal pulmonary infections caused by two prototypic MDR GNBs. In a validated mouse model, mAb H4 afforded >60% survival to infection by KP-QR as well as HUMC-1, as compared with control IgM having a 20% survival rate. Moreover, the mAb H3 provided 80–100% survival protection to mice from either of these GNBs, similar to that conferred by pAb (16). The efficacy seen by both H3 and H4 mAbs is afforded at the low concentration of 30 μg/mouse. This low dose of the mAb is about 1.2 mg/kg, which is within the dosage range of 1–15 mg/kg of most mAbs approved for human use (41, 42). This efficacy suggests that the mAbs likely neutralize functions of specific targets on the bacteria and attenuate their ability to exert virulence mechanisms or to cause disease in the host. This hypothesis is further supported by the observation that treatment with mAbs significantly mitigated dissemination of KP-QR and HUMC-1 to distal target organs within 2–4 days of treatment, vs. mice treated with control antibodies. These results provide compelling evidence of the robustness of the antibodies in abrogating pathogenesis, early in the onset of infection as well as in the setting of established infection.

In addition to specificity and safety, a key advantage of using mAbs as anti-infective therapy is their well-documented long half-life, which can exceed 21 days (43, 44). Because the patient population at risk of developing infections with *Acinetobacter, Klebsiella*, and *Candida* are well-defined, this property of mAbs may afford an extended protection during the time span of the greatest vulnerability. In turn, prevention of such infections would translate to reduced use of antibiotics and hence a reduced pressure for the emergence of drug resistance. For example, mAbs can be used to prophylax patients at risk of MDR GNBs. Another envisioned usage of these mAbs is their administration as adjunctive therapy with antibiotics. In this respect, we have demonstrated synergy of anti-peptide #5 pAb with imipenem or with colistin in killing *A. baumannii* at reduced minimum inhibitory concentration (MIC) of both antibiotics (16). Similarly, the anti-peptide #5 pAb synergistically acted with colistin in abrogating biofilm growth of *A. baumannii* (16).

In theory, one caveat for using novel Abs to treat infections caused by organisms known to develop antimicrobial resistance is the potential development of resistance to these Abs. However, cross-resistance between small molecule antimicrobials and antibacterial mAbs is unlikely because of the distinct therapeutic targets and pharmacological mechanisms that antibodies have as compared with traditional antimicrobials (45). In concept, other potential bacterial defense mechanisms could occur, such as synthesis of antibody-neutralizing proteins (e.g., protein A of *S. aureus*, which binds antibody Fc domain and prevent opsonophagocytosis (46)), or proteases to degrade the administered mAb (47, 48). However, none of the antibodies approved for treating infectious diseases [currently, there are only three Food and Drug Administration (FDA)-approved mAb to treat inhalational anthrax (49, 50) or *Clostridium difficile* (42) have encountered this issue, and development of resistance has not been reported.

In summary, we have demonstrated that mAbs raised against peptide #5 of Hyr1 target *A. baumannii* and *K. pneumoniae* and disrupt their ability to damage to host cells *in vitro*. More importantly, these mAbs protect mice from lethal pulmonary infections mediated by two high-priority GNBs. Thus, such mAbs have credible potential for development as prophylactic or adjunctive therapy to prevent or treat life-threatening infections in patients susceptible to MDR *A. baumannii* or *K. pneumoniae*.

## Materials and Methods

### Bacterial Strains and Growth Conditions

The bacterial strains used in this study are clinical isolates collected from Harbor-UCLA Medical Center (Torrance, CA). *Acinetobacter baumannii* strains HUMC-1 and HUMC-6 were separated from patients' sputum, and HUMC-12 was separated from a patient's wound and are XDR to all antibiotics, except colistin and tigecycline. *Klebsiella pneumoniae* strains were categorized into KPC or non-KPC isolates. The KPC-RM, KPC-6, and KPC-8 isolates resistant to carbapenem antibiotics possess *bla* KPC plasmid gene and separated from patients' sputum, whereas KP-QR is a non-KPC but multidrug-resistant isolate separated from a patient's sputum and resistant to gentamicin, kanamycin, and ampicillin/sulbactam antibiotics. All bacteria were cultured in tryptic soy broth (TSB) overnight at 37°C with shaking at 200 rpm. To obtain a log-phase bacterial suspension, overnight cultured bacteria were passaged in a fresh media (1:100) at 37°C with shaking for 3 h or until the cell concentration reached an OD_600_ of 0.5 (~2 × 10^8^ cells/ml) for both *A. baumannii* and *K. pneumoniae* isolates. The bacteria were diluted to the desired concentration from this stock.

### Computational Modeling of Structural Homology

Our previously validated Hyr1 model (16) was used as a template to seek structural homologs in *K. pneumoniae* having predicted epitopes for cross-kingdom immune protection. The Phyre 2.0 (51) and iTasser (52) platforms were used to generate homology models for Hyr1 and putative-related proteins. Results were scored based on 3-D threading homology and sequence relatedness and were integrated to identify conserved structural domains. As a confirmatory measure, additional stochastic modeling was carried out using the Quark server (53). Select regions of resulting comparative homologs were then subjected to 3-D alignment to identify areas of greatest homology using the Smith–Waterman (54) algorithm as implemented within Chimera (55). Sequence alignments to identify putative shared epitopes between Hyr1 and other proteins were carried out using CLUSTALW (56).

### Generation of Monoclonal Antibodies

Thirty micrograms of rHyr1 peptide #5 (synthesized by ProMab Biotechnologies, Richmond, CA) in 1 mg/ml of alum was used to immunize Balb/c mice (*n* = 10). The mice were boosted two times every 2 weeks with the same antigen concentration. Two weeks after the last boost, antibody titer was determined by ELISA plates coated with rHyr1 peptide #5. The spleens were collected, and the splenocytes were fused with hypoxanthine-guanine phosphoribosyltransferase (HGPRT)-negative murine myeloma cells at ratio 5:1 by slowly adding polyethylene glycol (PEG) to the cells pellet followed by adding Protein Free Hybridoma Media (PFHM) (Gibco, 12040077) supplemented with 20% heat-inactivated fetal bovine serum (FBS) (Corning, 35-016-CV). The cells were spun and re-suspended in 20% FBS PFHM and then incubated in 24-well plate at 37°C with 5% CO_2_ for 48 h. The media were replaced with hypoxanthine–aminopterin–thymidine (HAT) selection media for 8 days and then 20% FBS PFHM hypoxanthine-thymidine (HT) media for 2 weeks. The grown hybridoma clones were diluted by microdilution in microtiter plates to achieve one cell per well and propagated in 10% FBS PFHM. The supernatant from the grown clones was tested for anti-Hyr1 antibodies using ELISA. The selected positive and stable clones were cultured in PFHM without FBS, and the cell numbers were adjusted to be 2 × 10^5^/ml for optimum production of mAbs.

### Detoxification and Purification of the Supernatant Containing the Monoclonal Antibodies

The supernatant containing the antibodies was concentrated using 100-kDa cutoff centrifugal concentrating tube (Amicon, UFC910024). HiTrap HP column (GE Healthcare, 17511001) was used to purify the concentrated mAbs and then was buffer exchanged with endotoxin-free Dulbecco phosphate-buffered saline (PBS) without calcium or magnesium (Gibco, 14190250). Endotoxin was tested using a chromogenic limulus amebocyte lysate assay (BioWhittaker Inc.), and all mAbs had low-range endotoxin level of <0.015 EU/ml. The isotype of the mAbs was identified using ELISA and confirmed by molecular weight using sodium dodecyl sulfate–polyacrylamide gel electrophoresis (SDS-PAGE).

### Surface Staining and Binding Assay

Bacterial cells (5 × 10^6^ cells) re-suspended in 2% FBS–PBS were incubated with anti-Hyr1 mAbs or isotype-matched IgM control (BD Biosciences) for 2 h at a range of concentrations (100–0.1 μg/ml). The bacterial cells were washed three times with cold 2% FBS–PBS. The bound anti-Hyr1 mAbs to the bacterial cells were detected with anti-mouse FITC-labeled secondary antibodies (Thermo Fisher Scientific). The unbound antibodies were washed three times with cold 2% FBS–PBS before the measurement of the fluorescent-stained bacterial cells using flow cytometry (Becton Dickinson FACSCalibur), where it is adjusted to detect up to 20,000 events per sample.

### Cell Damage Assay

The *in vitro* ability of mAbs to protect either A549 cells or HUVECs from damage caused by direct contact with bacteria was measured using ^51^Cr release assay, modified from previous method (57). Isolation of HUVECs was performed in the laboratory under a protocol approved by institutional review board (IRB). Because umbilical cords are collected without donor identifiers, our IRB considers them medical waste and not subject to informed consent.

Alveolar epithelial A549 cells and HUVECs were incubated overnight in 24-well plates with F-12K or Roswell Park Memorial Institute (RPMI1640) medium supplemented with 10% FBS, containing 1 μCi/well of Na_2_^51^CrO_4_ (ICN Biomedicals, Irvine, CA). The next day, unincorporated tracer was aspirated, and the wells were rinsed three times with warm Hanks' Balanced Salt Solution (HBSS). One milliliter of media containing HUMC-1 or KP-QR (pre-incubated with mAbs or IgM isotype control for 1 h on ice) was then added to host cells in each well at a multiplicity of infection (MOI) of 1:100 (host cells to bacteria), and the plate was incubated for 48 or 24 h, respectively, at 37°C in 5% CO_2_. At the end of the incubation, all the media were gently aspirated from each well, after which the mammalian cells were lysed by the addition of 0.5 ml of 6 N NaOH. The lysed cells were aspirated, and the wells were rinsed twice with RadioWash (Atomic Products, Inc., Shirley, NY). These rinses were added to the lysed cells, and the ^51^Cr radioactivity of the medium and the cell lysates was determined. Control wells containing media but no organisms were processed in parallel to measure the spontaneous release of ^51^Cr. After corrections were made for the differences in the incorporation of ^51^Cr in each well, the specific release of ^51^Cr was calculated by the following formula: (experimental release–spontaneous release)/(total incorporation–spontaneous release).

### Animal Models

Male CD-1 immunocompetent mice (4–6 weeks old) were used for *Klebsiella* (KP-QR) intratracheal infection or immunosuppressed mice infected with *A. baumannii* (HUMC-1) by an aerosolization chamber to induce pneumonia by inhalation. Mice were immunosuppressed by administrating cyclophosphamide (200 mg/kg) (i.p.) and cortisone acetate (250 mg/kg) (subcutaneous) on Days −2, +3, and +8 relative to infection as previously described Gebremariam et al. (57). A total of 30 μg/mouse of mAbs or isotype-matched control were administrated (i.p.) on Day +1 and on Day +4 post infection. Survival of mice served as an endpoint. For quantitative measurement of bacterial burden, mAbs were administered 6 h after infection, and a repeat dose was given on Day +3. Mice were euthanized on Day +4 for *A. baumannii* and on Day +2 for *K. pneumoniae*. Lungs were harvested aseptically and homogenized, and the bacterial burden was determined by quantitative culturing on tryptic soy agar plates.

### Statistical Analysis

The percentage of cell damage and tissue bacterial burden was compared using non-parametric Mann–Whitney test. The log-rank test was used to determine the difference in survival studies. *P* < 0.05 was considered significant.

## Data Availability Statement

All datasets generated for this study are included in the article/Supplementary Material.

## Ethics Statement

All procedures involving mice were approved by the Institutional Animal Care and Use Committee (IACUC) of the Lundquist Institute for Biomedical Innovation at Harbor-UCLA Medical Center (protocol number 20295), according to the National Institutes of Health (NIH) guidelines for animal housing and care. Moribund mice according to detailed and well-characterized criteria were euthanized by pentobarbital overdose, followed by cervical dislocation.

## Author Contributions

EY performed conceptualization, data curation, formal analysis, investigation, methodology, and writing–original draft. SA, TG, and LZ performed investigation and methodology. SS performed data analysis and manuscript revision. NY performed methodological and data interpretation. MY performed conceptualization, methodology, data interpretation, and manuscript revision. PU performed formal analysis, investigation, methodology, and writing–original draft. AI performed conceptualization, data curation, formal analysis, funding acquisition, investigation, methodology, project administration, supervision, and manuscript revision.

### Conflict of Interest

MY and AI are founders and shareholders of NovaDigm Therapeutics, Inc., which is developing novel immunotherapies targeting priority pathogens. The remaining authors declare that the research was conducted in the absence of any commercial or financial relationships that could be construed as a potential conflict of interest.
