# Supplementary material for: Monoclonal IgM Antibodies Targeting Candida albicans Hyr1 Provide Cross-Kingdom Protection Against Gram-Negative Bacteria
Source: Front Immunol. 2020 Feb 18;11:76. doi: 10.3389/fimmu.2020.00076 (PMC7045048; doi:10.3389/fimmu.2020.00076)
Supplement: Supplementary file 1 [file Presentation_1.PPTX]

## Slide 1
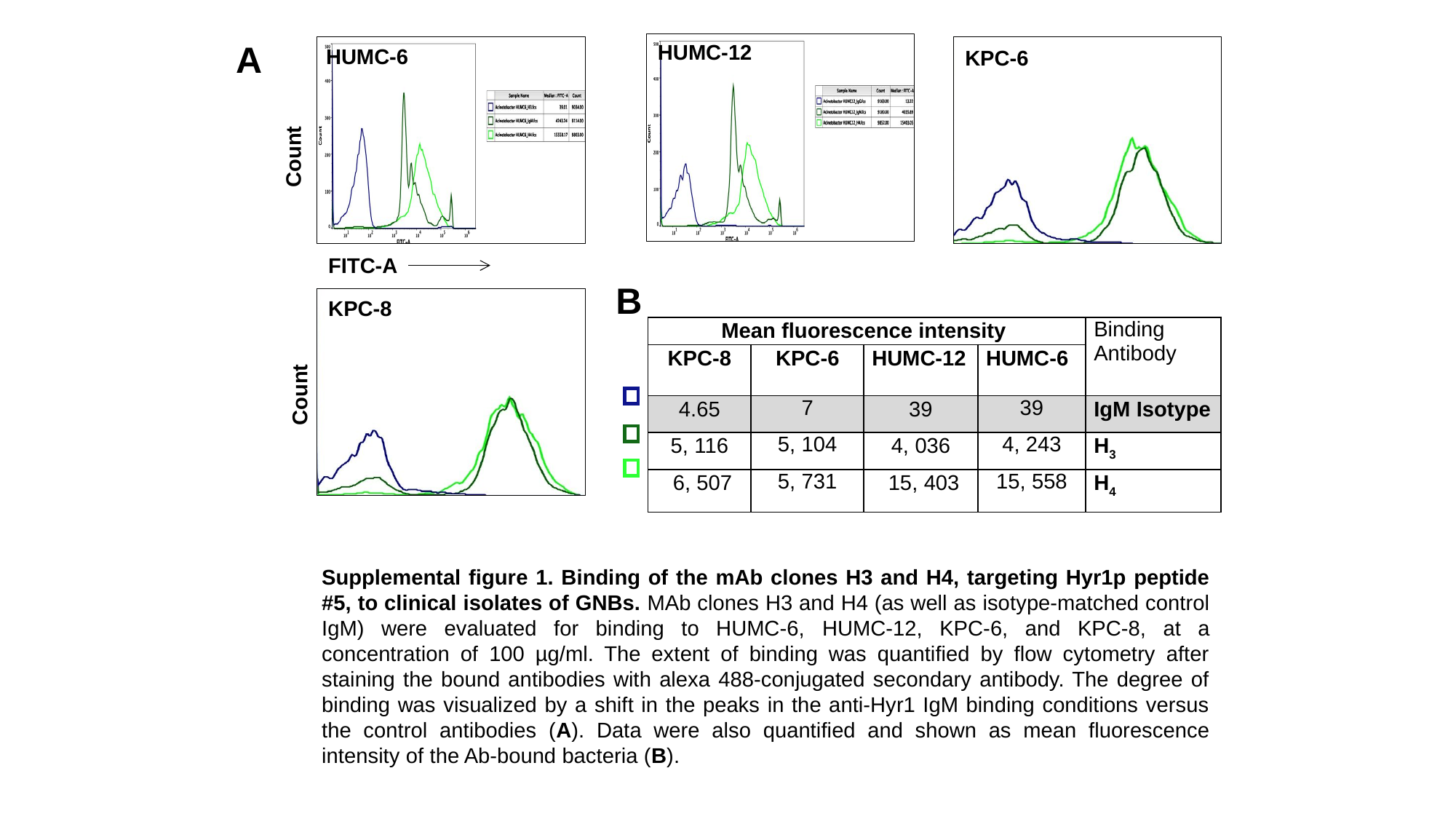

A
HUMC-12
HUMC-6
KPC-6
Count
FITC-A
B
KPC-8
| Mean fluorescence intensity | | | | Binding Antibody |
| --- | --- | --- | --- | --- |
| KPC-8 | KPC-6 | HUMC-12 | HUMC-6 | |
| 4.65 | 7 | 39 | 39 | IgM Isotype |
| 5, 116 | 5, 104 | 4, 036 | 4, 243 | H3 |
| 6, 507 | 5, 731 | 15, 403 | 15, 558 | H4 |
Count
Supplemental figure 1. Binding of the mAb clones H3 and H4, targeting Hyr1p peptide #5, to clinical isolates of GNBs. MAb clones H3 and H4 (as well as isotype-matched control IgM) were evaluated for binding to HUMC-6, HUMC-12, KPC-6, and KPC-8, at a concentration of 100 µg/ml. The extent of binding was quantified by flow cytometry after staining the bound antibodies with alexa 488-conjugated secondary antibody. The degree of binding was visualized by a shift in the peaks in the anti-Hyr1 IgM binding conditions versus the control antibodies (A). Data were also quantified and shown as mean fluorescence intensity of the Ab-bound bacteria (B).

## Slide 2
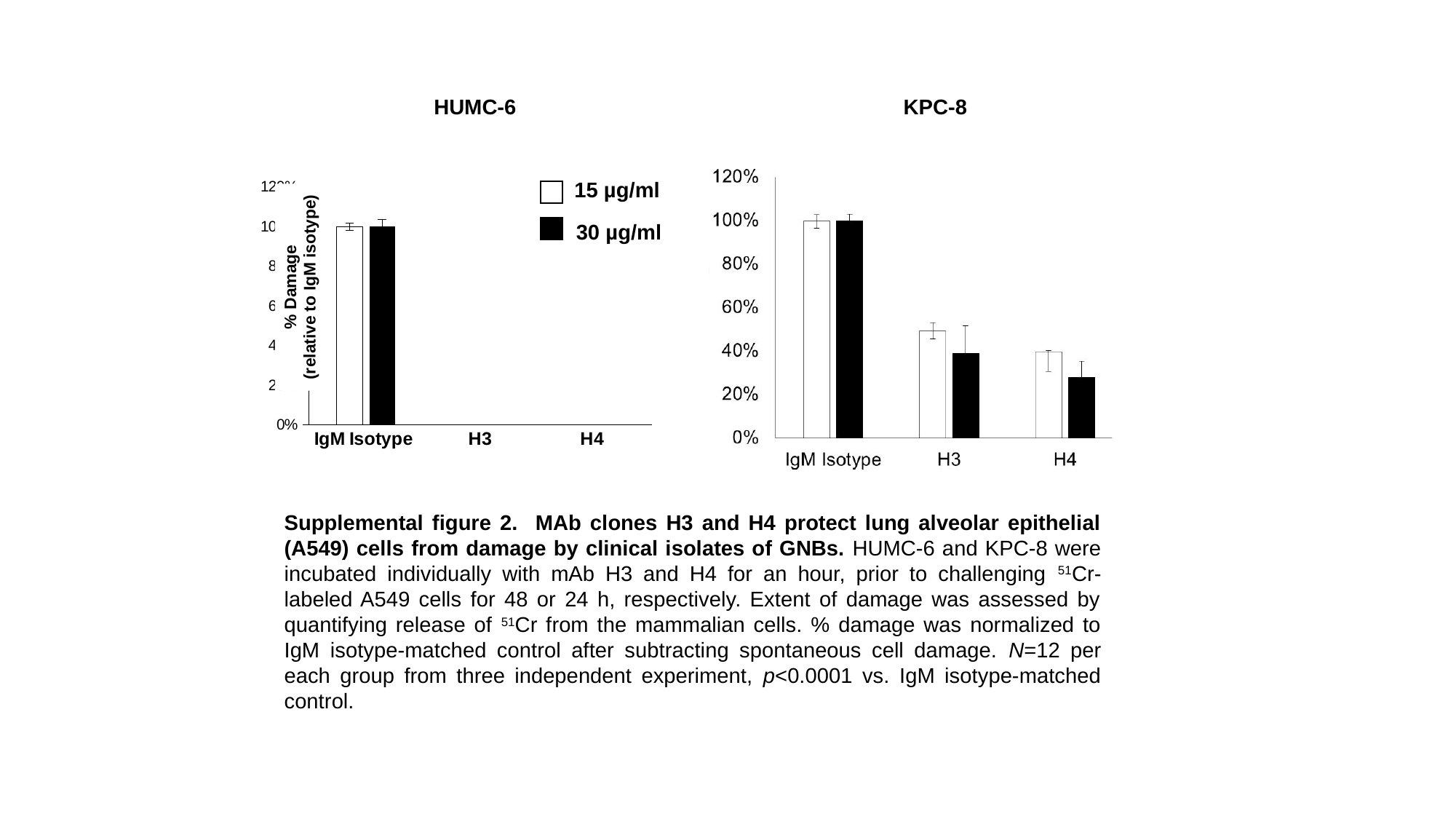

### Chart
| Category | | |
|---|---|---|
| IgM Isotype | 1.0 | 1.0 |
| H3 | -0.2695362631913548 | -0.29070901606798166 |
| H4 | -0.29457984286005884 | -0.4462152833409958 |KPC-8
HUMC-6
15 µg/ml
30 µg/ml
Supplemental figure 2. MAb clones H3 and H4 protect lung alveolar epithelial (A549) cells from damage by clinical isolates of GNBs. HUMC-6 and KPC-8 were incubated individually with mAb H3 and H4 for an hour, prior to challenging 51Cr-labeled A549 cells for 48 or 24 h, respectively. Extent of damage was assessed by quantifying release of 51Cr from the mammalian cells. % damage was normalized to IgM isotype-matched control after subtracting spontaneous cell damage. N=12 per each group from three independent experiment, p<0.0001 vs. IgM isotype-matched control.

## Slide 3
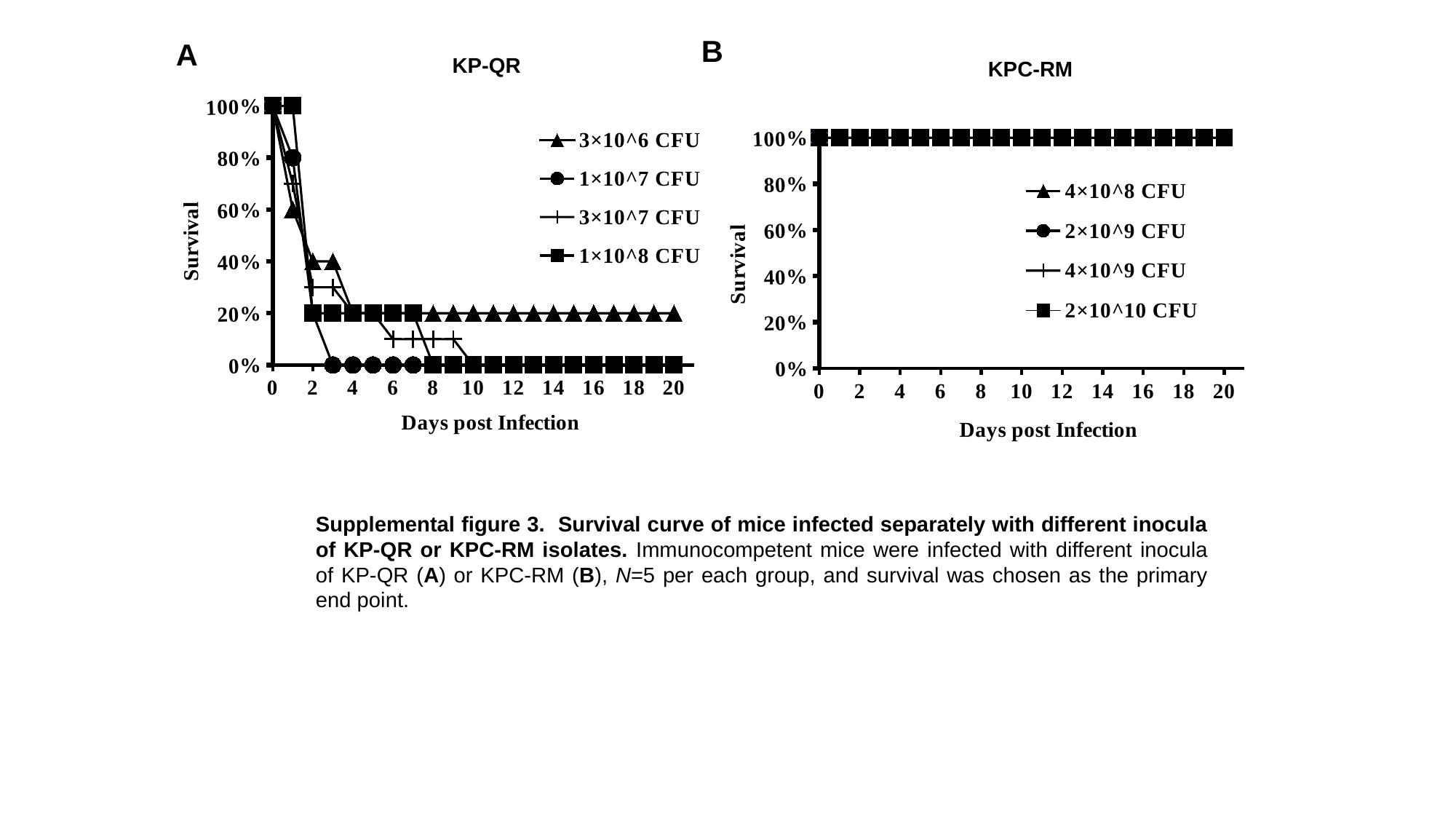

B
A
KP-QR
KPC-RM
### Chart
| Category | 3×10^6 CFU | 1×10^7 CFU | #REF! | #REF! | #REF! | #REF! | 3×10^7 CFU | 1×10^8 CFU |
|---|---|---|---|---|---|---|---|---|
### Chart
| Category | 4×10^8 CFU | 2×10^9 CFU | #REF! | #REF! | #REF! | #REF! | 4×10^9 CFU | 2×10^10 CFU |
|---|---|---|---|---|---|---|---|---|Supplemental figure 3. Survival curve of mice infected separately with different inocula of KP-QR or KPC-RM isolates. Immunocompetent mice were infected with different inocula of KP-QR (A) or KPC-RM (B), N=5 per each group, and survival was chosen as the primary end point.
